# Prospective, observational practice survey of applied skin care and management of cetuximab-related skin reactions: PROSKIN study

**DOI:** 10.1007/s00280-019-03927-x

**Published:** 2019-08-23

**Authors:** Sacha I. Rothschild, Daniel Betticher, Reinhard Zenhäusern, Sandro Anchisi, Roger von Moos, Miklos Pless, Peter Moosmann, Razvan A. Popescu, Antonello Calderoni, Marco Dressler, Daniel Rauch, Stefanie Pederiva, Regina Woelky, Claudia Papet, Vera Bühler, Markus Borner

**Affiliations:** 1 Departement Innere Medizin, Medizinische Onkologie, Universitätsspital Basel, Petersgraben 4, 4031 Basel, Switzerland; 2 HFR Fribourg, Hôpital fribourgeois, Fribourg, Switzerland; 3 Spital Wallis, Spitalzentrum Oberwallis, Brig, Switzerland; 4 Hôpital du Valais, CHVR, Sion, Switzerland; 5 Kantonsspital Graubünden, Medizinische Onkologie, Chur, Switzerland; 6 Kantonsspital Winterthur, Tumorzentrum, Winterthur, Switzerland; 7 Kantonsspital Aarau, Medizinische Onkologie, Aarau, Switzerland; 8 Hirslanden Tumor Zentrum Aarau, Aarau, Switzerland; 9 Oncologia Varini Calderoni Christinat, Lugano, Switzerland; 10 Hirslanden Klinik St. Anna, Luzern, Switzerland; 11 Spital Thun, Medizinische Onkologie, Thun, Switzerland; 12 Kantonsspital Baden, Onkologie Standort Brugg, Brugg, Switzerland; 13 Kantonsspital Frauenfeld STGAG, Medizinische Onkologie, Frauenfeld, Switzerland; 14 Limmattalspital, Medizinische Onkologie, Schlieren, Switzerland; 15 Merck (Schweiz) AG, Zug, Switzerland; 16 Spitalzentrum Biel, Medizinische Onkologie, Biel, Switzerland

**Keywords:** Cetuximab, Observational, Practice survey, Management, Skin reactions

## Abstract

**Purpose:**

The study aimed to investigate strategies to prevent and treat cetuximab-induced skin reactions and their perceived effectiveness in patients with metastatic colorectal cancer (mCRC) and recurrent/metastatic squamous cell cancer of the head and neck (SCCHN).

**Methods:**

This open-label, prospective observational study was conducted in Switzerland.

**Results:**

A total of 125 patients were included (*n* = 91 mCRC, *n* = 34 SCCHN; mean age 63.3 years; 73.6% males). The frequency of acneiform rash grade ≥ 2 increased from 12.6% at week 2 to 21.7% at week 16. The proportion of patients who reported no skin reaction decreased from 75.6% at week 2 to 43.3% at week 16. The most frequently used skin products at any time of observation were moisturizing (77.6%), lipid-regenerating (56.8%) or urea-containing products (52%), systemic antibiotics (49.6%), and vitamin K1 cream (43.2%). There was no clear effectiveness pattern for all product classes: in given patients, either the product showed no effect at all or a moderate/strong effect, consistently over time.

**Conclusions:**

A great variety of low-cost general skin care products were commonly used. According to physician’s preference, systemic antibiotics and vitamin K1 cream are an appropriate approach to prevent or treat cetuximab-related skin toxicity.

## Introduction

Cetuximab is a chimeric monoclonal antibody that binds and inactivates the epidermal growth factor receptor (EGFR). The mechanism of blocking EGFR is an important strategy in the treatment of malignancies of epithelial origin such as colorectal cancer and squamous cell cancer of the head and neck [1, 2]. While this therapeutic approach is usually better tolerated than conventional chemotherapy, it has a unique side-effect profile related to the mechanism of action. EGFR is not solely expressed on tumor cells, but also on cells of the epidermis. Thus, skin toxicities, including rash, pruritus, dry skin, desquamation, hypertrichosis, and nail disorders, are seen in approximately 80% of patients treated with cetuximab [3–5]. Unless properly managed, these can result in dose reductions and discontinuation of treatment, in about 15–25% of patients [6–8]. The most common (90% of patients with cutaneous toxicity) and clinically most relevant skin toxicity associated with cetuximab is the papulopustular rash, also called acneiform rash [9]. Dermatologic toxicities are rarely life threatening; however, they impair quality of life (QoL) and treatment compliance. Therefore, an effective management of skin toxicities is crucial to maximize treatment efficacy and maintain QoL.

The cetuximab prescribing information states that skin reactions are very common and may require treatment interruption or discontinuation [10]. It recommends that according to clinical practice guidelines, prophylactic use of oral tetracyclines (6–8 weeks) and topical application of 1% hydrocortisone cream with moisturizer should be considered. Medium- to high-potency topical corticosteroids or oral tetracyclines have been used for the treatment of skin reactions [10, 11].

In daily clinical practice, multiple care and management options to reduce the severity of skin reactions are used, including but not limited to topical or oral antibiotics, or glucocorticosteroids. Antihistamines and local anesthetics can be administered to reduce pruritus. The frequent use of cetuximab in cancer therapy and the lack of specific clinical studies necessitate the need for studies evaluating the measures taken to alleviate skin reactions in patients treated with cetuximab.

The current observational study was initiated to gain information about the perceived effectiveness of the measures taken to alleviate skin reactions in cetuximab-treated patients. Furthermore, we aimed at assessing the impact of skin reactions on the treatment course, QoL and the reason of physicians’ choice for specific therapies.

## Methods

### Design

PROSKIN is a prospective observational study with a nonexperimental cohort design to provide insight into the currently used prophylactic measures and management strategies of cetuximab-related skin reactions in patients with metastatic colorectal cancer (mCRC) and or recurrent or metastatic (r/m) squamous cell cancer of head and neck (SCCHN) and the perceived effect of these strategies.

### Sites

Twenty-three sites in Switzerland that were experienced in the management of tumor patients actively enrolled patients. Data collection was performed between 03 October 2012 and 30 April 2016.

### Patients

Patients with mCRC or r/m SCCHN who received at least one dose of cetuximab were eligible for this study. All patients gave written informed consent prior to the study. The study was approved by local ethical committees.

The following exclusion criteria applied: (1) current radiotherapy; (2) pre-existing skin reaction (acneiform rash, dryness of skin, pustule formation, pruritus, erythema); (3) patients not willing to respond to questions from the physician; (4) patients not suitable to receive cetuximab according to the summary of product characteristics; (5) legal incapacity or limited legal capacity; (6) any psychological or medical condition that would not permit a meaningful signature of the informed consent.

In this study, the only AEs that were to be reported in the eCRF were those AEs affecting the skin. In addition, SAEs were to be reported to the Merck Global Patient Safety database, which are also reported here.

### Statistical considerations

Primary data sources were the patient’s medical records and online electronic case report forms (eCRFs) completed at the time of consultation.

Descriptive statistics were provided for all continuous variables. 95% confidence intervals (CI) were provided wherever applicable. In terms of sample size, it was planned for approximately 200 patients to be documented in the study.

The Full Analysis Set (FAS) included patients diagnosed with mCRC or r/m SCCHN who received at least one cycle of cetuximab and data regarding skin care products and perceived effectiveness of those products were reported. The Safety Set contains all patients for whom cetuximab therapy was started.

Patients were assessed at baseline and weeks 2, 4, 6, 10 and 16. Questionnaires were completed by patients at the day of clinical visit and appointment with the treating physician at weeks 2, 4, 6, 10 and 16. The maximal observation time was 16 weeks.

The primary end point was physician’s perceived effectiveness of the skin products (skin care and medication) used.

Statistical analysis was performed using Statistical Analysis System (SAS) Version 9.1.3. (NC, USA).

## Results

### Patient disposition

**Fig. 1:**
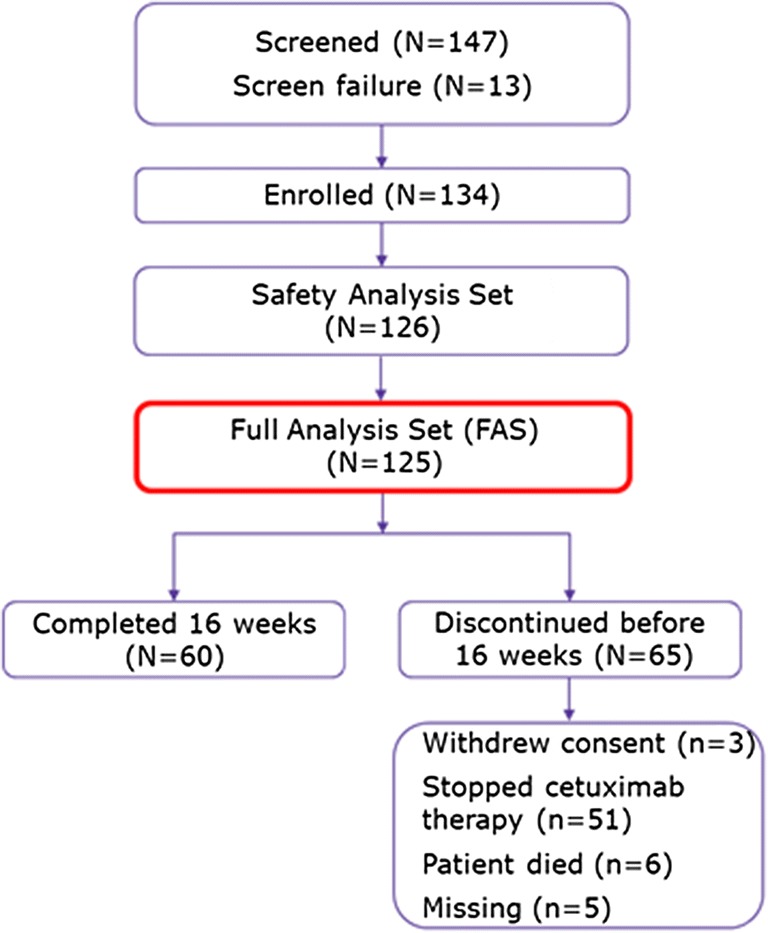
Patient disposition. Of the enrolled 134 patients, 9 (6.7%) patients were not included in the FAS due to unknown diagnosis or a diagnosis other than mCRC or r/m SCCHN (*n* = 4) or no cetuximab treatment documented (*n* = 5).126 started cetuximab treatment. 125 patients (FAS) were evaluable

Patient disposition is shown in Fig. 1. A total of 134 patients were enrolled.

The study stopped prematurely due to slow accrual. Of the enrolled 134 patients, 9 (6.7%) were not included in the FAS due to unknown diagnosis or a diagnosis other than mCRC or r/m SCCHN (*n* = 4) or no cetuximab treatment documented (*n* = 5). In total, 126 patients were analyzed in the Safety Set and 125 in the Full Analysis Set (FAS). One out of the 126 experienced an adverse infusion-related reaction during the first cetuximab administration and was removed from the study. This patient was excluded from the FAS, because no data regarding skin care products and perceived effectiveness of those products were reported. While 60 patients (48.0%) completed the study (16 weeks), 65 patients (52.0%) discontinued prematurely. This was mainly due to cessation of cetuximab therapy (*n* = 51) or due to death (*n* = 6), missing information (*n* = 5) or withdrawal of consent (*n* = 3).

### Demographics, baseline characteristics and planned treatment

Baseline characteristics of patients in the FAS are shown in Table 1. Mean age of patients was 63.3 ± 11.4 (range 29–84 years), and 73.6% were men. Ninety-one patients (72.8%) were diagnosed with mCRC and 34 (27.2%) with r/m SCCHN.

**Table 1:** Baseline demographics and medical history

Characteristic (FAS, *N *= 125)	*n*	Value
Demographics		
Age, mean ± SD, years	125	63.3 ± 11.4
Min; Max		29; 84
Sex, males, %	92	73.6
Females, %	33	26.4
Body surface area, m^2^	125	1.80 ± 0.20
Medical history		
Metastatic colorectal cancer	91	72.8
Recurrent/metastatic squamous cell cancer of the head and neck	34	27.2
ECOG performance score		
0	61	48.8
1	54	43.2
2	10	8.0
3	0	0.0
4	0	0.0
Missing	0	0.0
Previous anti-cancer treatment^a^		
Surgery	75	60.0
Chemotherapy	58	46.4
Radiotherapy	34	27.2
Biologic	19	15.2
None	23	18.4

*FAS* Full Analysis Set, *SD* standard deviation

^a^Multiple responses were present in the data per patients

The Eastern Cooperative Oncology Group (ECOG) performance score of the majority of patients was either 0 (*n* = 61, 48.8%) or 1 (*n* = 54, 43.2%). Cetuximab was used as first-line treatment in 77 patients (61.6%), as second-line treatment in 31 patients (24.8%), as third-line in 13 patients (10.4%) and later lines in 3 patients (2.4%).

### Prophylaxis and treatment

Table 2 lists the skin products used (skin care products and medications administered to treat or prevent skin reactions) at any time during the study. The most frequently used skin products were moisturizing agents (97 patients, 77.6%), lipid-regenerating products (71 patients, 56.8%), urea-containing products (65 patients, 52.0%), systemic antibiotics (62 patients, 49.6%) and vitamin K1 cream (54 patients, 43.2%).

**Table 2:** Use of skin care products and medications

Planned treatment (FAS, *N *= 125)	*n*	%	Prophylactic	Reactive
Moisturizing products	97	77.6	82	33
Lipid-regenerating products	71	56.8	51	24
Urea-containing products	65	52.0	57	20
Systemic antibiotics	62	49.6	35	32
Vitamin K1 cream	54	43.2	42	25
Systemic antihistamines	36	28.8	30	5
Systemic steroids	28	22.4	23	3
Topical steroids aseptic products	In about 25%		8 18	20 8
Wet wraps, other topical treatments, topical antibiotics, other systemic treatments, combinations of topical antibiotics + topical steroids	In 4–12%			

*FAS* Full Analysis Set

Other medications included topical steroids, antiseptic products, wet wraps, other topical treatments, topical antibiotics, other systemic treatments, topical antibiotics plus topical steroids combined in one product. These products were used in less than 30% of patients.

Systemic steroids (28% of patients) and systemic antihistamines (28% of patients) were used as pre-medications prior to cetuximab infusions, and as treatment of infusion-related reactions.

### Effectiveness

#### Categorical effectiveness

**Fig. 2:**
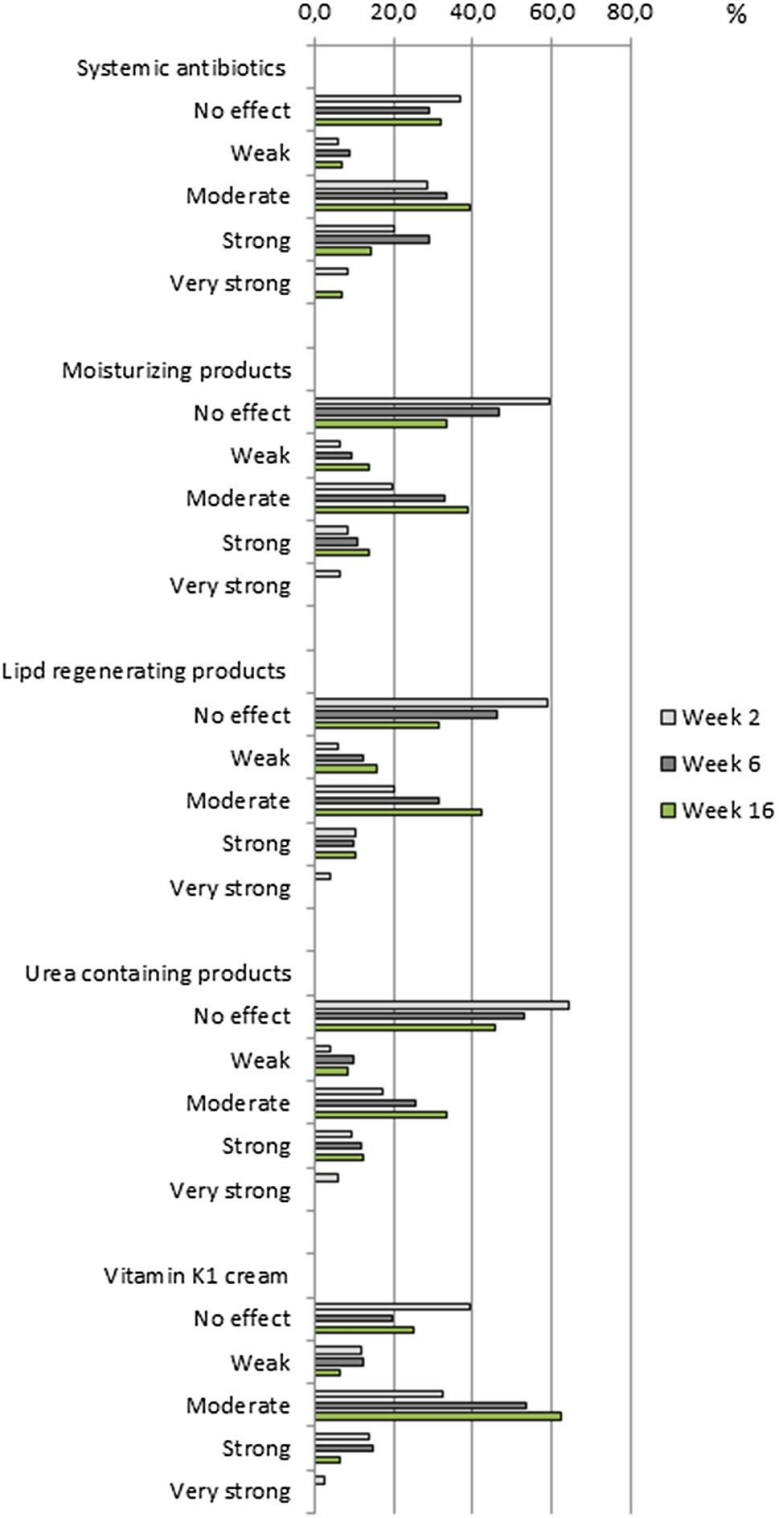
Physicians’ perceived effectiveness of skin care and medication, categorical. FAS *n* = 125. Primary end point denominator for % calculation was the total number of patients in FAS who received the medication/skin care product at the time point specified. *FAS* Full Analysis Set

The perceived effectiveness of the skin products (primary end point) is summarized for the most frequently administered agents at weeks 2, 6 and 16 in Fig. 2.

For all drug classes, effectiveness ratings varied across patients: “no effect” and “moderate”/“strong” were the preferred ratings of physicians and the two peaks stayed over the time. “Weak” or “very strong” was rarely mentioned. For example, “moderate” to “very strong” effectiveness was perceived by a majority of physicians in patients who received systemic antibiotics at week 2 (57.2% of 35 patients treated) and at week 6 (62.2% of 45 patients treated). This frequency remained at the same level until week 16 (60.7% in 28 patients treated by week 16). Overall, the percentages of responses “no effect” lowered and “moderate” gained percentages over time.

#### Mean effectiveness across visits

**Fig. 3:**
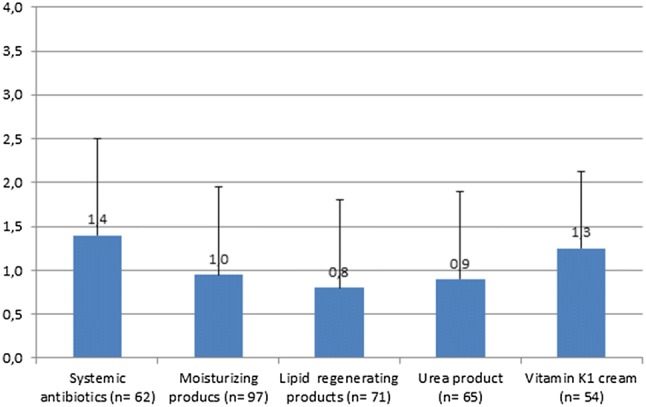
Physicians’ perceived effectiveness of skin care and medication, mean. Average of the assessments across all time points for each patient and type of medication using efficacy value as 0 = no, 1 = weak, 2 = moderate, 3 = strong and 4 = very strong. Prophylactic and therapeutic use are combined. Denominator for % calculation was the total number of patients in FAS. *FAS* Full Analysis Set. Whiskers represent standard deviation

On calculation of the average numerical effectiveness values (from 0 = no, to 4 = very strong) of the assessments across all visits for each patient and for each type of medication, mean perceived effectiveness (regardless of prophylactic or reactive usage) was highest for the combination of topical antibiotics and steroids (1.95 ± 1.16 in 14 patients), followed by systemic antibiotics (1.40 ± 1.10 in 62 patients) and vitamin K1 cream (1.25 ± 0.87 in 54 patients, Fig. 3). Lowest mean effectiveness values were observed for antiseptic products (0.67 ± 0.98 in 26 patients), and lipid-regenerating products (0.83 ± 1.00 in 71 patients), respectively.

The average values for reported pre-medications, regardless of prophylactic or reactive usage was highest for antihistamines (1.81 ± 1.36 in 36 patients), followed by systemic steroids (1.72 ± 1.37 in 28 patients).

#### Impact of skin reactions on the course of therapy

No relevant differences, in the cetuximab dose (mg/m^2^) or the percentage of dose delays, were observed between patients who had the first occurrence of skin reactions early (i.e., at week 2, 4 or 6) in comparison to those with a first occurrence at later time points (weeks 10 or 16), or without any skin reaction. This was also the case for the first occurrence of skin reactions grade ≥ 2 or for the first occurrence of acneiform rash (both any grade and grade ≥ 2).

#### Patient’ impressions of skin reactions: itching intensity

A majority of the patients experienced ‘no itching’ at any time, i.e., 75.6% at week 2, 53.6% at week 4, 55.9% at week 6, 54.9% at week 10 and 53.3% at week 16. Strong or very strong intensity was reported in very few patients (3.4% at week 2, 1.0% at week 6 and none at week 16).

#### Impact on daily life

At week 2, 75.6% patients reported no impact of skin reactions on daily life. Thereafter the proportion of patients with no impact decreased to 57.1% at week 4, 52.9% at week 6, 41.5% at week 10 and 43.3% at week 16. Very strong impact on daily life was reported by very few patients (1.7% at week 2, 1.0% at week 6 and 1.7% at week 16).

#### Influence on willingness to continue therapy

A majority of the patients reported no influence of skin reactions on their willingness to continue therapy and the proportion of patients remained almost identical at all weeks (68.9% at week 2, 67.0% at week 4, 66.7% at week 6, 64.6% at week 10, and 63.3% at week 16).

At week 2, 21.0% of patients strongly favoured continuation of therapy. Thereafter, the proportion of patients slightly increased to 24.1% at week 4, 24.5% at week 6, 25.6% at week 10 and 28.3% at week 16. Very few patients (0.8% at week 2; 0.9% at week 4; 0% at weeks 6 and 10; 1.7% at week 16) strongly favoured discontinuation of therapy.

**Fig. 4:**
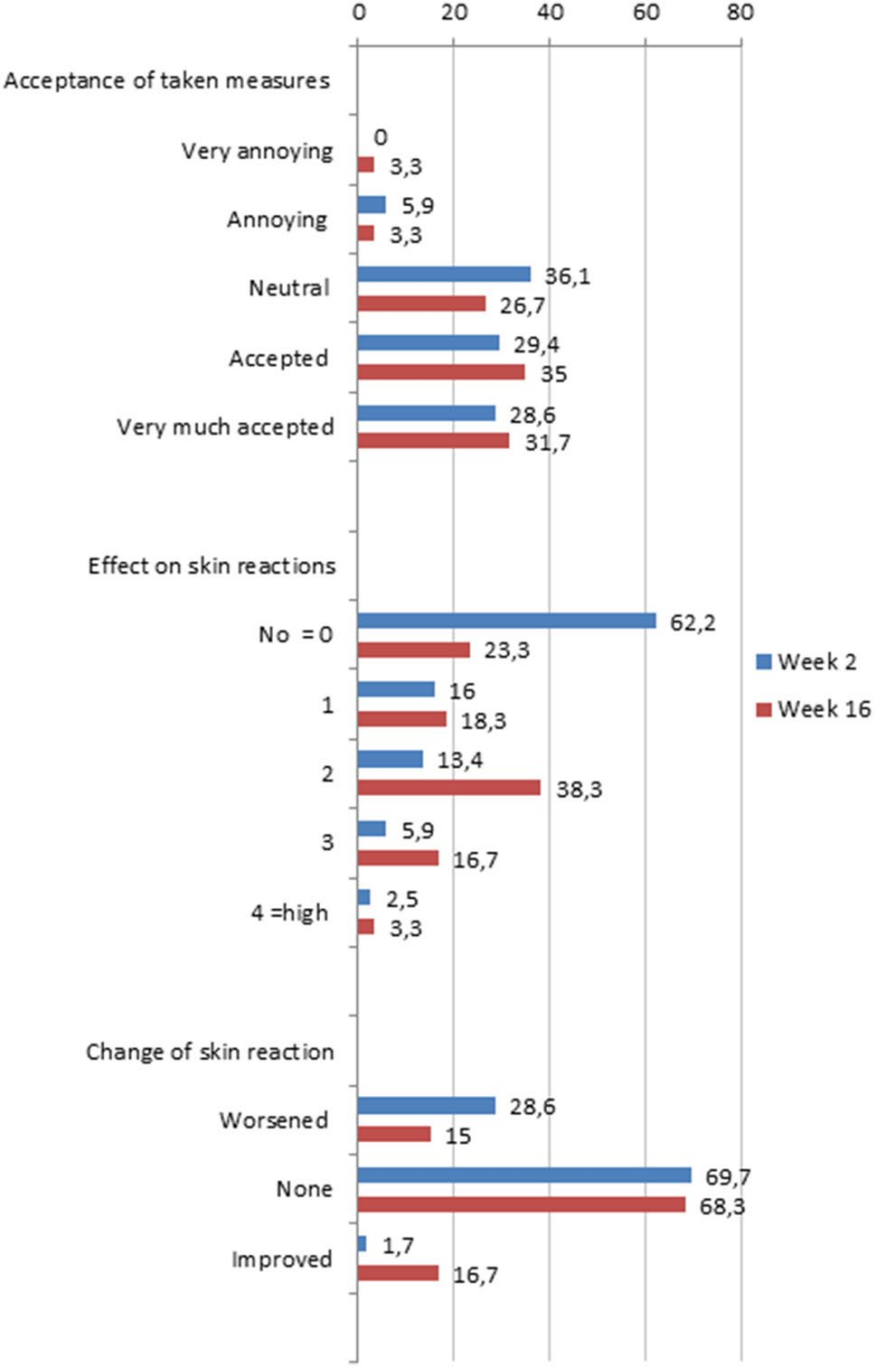
Patients’ perception of measures taken

Perceptions of the measures taken are summarized in Fig. 4.

#### Acceptance of measures

At week 2, 36.1% patients perceived the measures taken, against skin reactions, to be neutral, which slightly decreased thereafter (32.1% at week 4, 29.4% at week 6, 26.8% at week 10, and 26.7% by week 16). A similar proportion of patients at all weeks (28.6% at week 2, 33.0% at week 4, 32.4% at week 6, 32.9% at week 10, and 31.7% at week 16) ‘very much accepted’ the measures taken. Very few patients found the measures taken, against skin reactions, as very annoying (0.0% at week 2, 2.7% at week 4, 1.0% at week 6, 2.4% at week 10 and 3.3% at week 16).

#### Effect of skin care measures and medication on skin reactions

A majority of the patients reported no impact of skin care measures and medication on skin reactions at week 2 (62.2%). Thereafter, the proportion of patients with no impact decreased to 34.8% at week 4, 30.4% at week 6, 24.4% at week 10 and 23.3% at week 16. At week 6 and thereafter, a majority of patients reported a better than weak efficiency (53% at week 6, 58.5% at week 10 and 58.3% at week 16).

#### Change of skin reaction

A majority of the patients reported no change in the skin reaction at week 2 (69.7%). The proportion of patients reduced to 43.8% at week 4 and showed an increasing trend in later weeks (51.0% at week 6, 54.9% at week 10 and 68.3% at week 16). Only 1.7% patients at week 2 perceived an improvement in skin reactions. The proportion of patients increased to 12.5% at week 4, 24.5% at week 6 and later decreased to 15.9% patients at week 10. By 16 weeks, 16.7% patients perceived an improvement in skin reactions.

### Safety

#### Skin reactions incidence and grading

Acneiform rash occurred in 94 patients (74.6%), dryness of skin in 78 (61.9%), pruritus in 56 (44.4%), paronychia in 21 (16.7%) and erythema in 47 (37.3%). The majority of these reactions were grade 1 and to a lesser extent grade 2. There were no grade 4 reactions. Only one serious adverse event was reported as related to the skin (acneiform rash). In this case, rash, dyspnea, tachycardia and nausea occurred during the first infusion and were rated as IRR.

#### Premature discontinuations

The primary reasons for premature cessation of the study as reported for 65 patients were discontinuation of cetuximab. [51 cases, patient death (6 cases) and withdrawn consent (3 cases). The cause of death in five patients was the outcome of one or more SAEs, two of which were disease progression; the cause of death for one patient was unknown. The dropout information was missing in 5 patients.] Cessation of cetuximab therapy was related to skin reactions in five patients (4.0% of 125 patients treated).

#### Serious adverse events

During the course of the study, there were 26 SAES (excluding two events of disease progression) in 16 Individual Case Safety Reports (ICSR). Four of these 16 ICSRs reported 6 SAEs with a fatal outcome: pneumonia in 1 ICSR, gastric hemorrhage and vascular injury in 1 ICSR, infection in 1 ICSR, ischemic colitis and infectious colitis in 1 ICSR. The remaining 12 ICSRs reported 20 SAEs: rash, dyspnea, tachycardia and nausea in 1 ICSR (all events occurred during the first infusion and were regarded as IRR), fall, femoral neck fracture, foot fracture in 1 ICSR, nausea, vomiting, diarrhea and dehydration in 1 ICSR, hypersensitivity in 2 ICSRs, infusion-related reaction, anaphylactic shock, device-related infection, renal failure, deep vein thrombosis, dysphagia and diarrhea in 1 ICSR each.

## Discussion

The optimal approach on how to prevent and manage the cutaneous side effects of cetuximab and other EGFR-targeting antibodies has not been clearly established. The prospective observational PROSKIN study provides detailed insights into current preventive measures and treatment of skin reactions related to cetuximab in Switzerland. The study included patients from 23 sites in the country including university hospitals, secondary- and primary-level hospitals and oncological practices, therefore reflecting the full spectrum of oncological patient care in the country. The study demonstrates a broad variety of preventive and therapeutic skin care that is used in patients undergoing cetuximab therapy. Each patient receives on average three products at the same time. The most frequently used products in this study were moisturizing, lipid-regenerating and urea-containing products (i.e., no typical pharmaceutical products), followed by systemic antibiotics and vitamin K1 cream. Thus, physicians mainly used low-cost topical products.

Moderate to very strong effectiveness was perceived by a majority of physicians for patients who received systemic antibiotics or vitamin K1 cream, while moisturizing, lipid-regenerating and urea-containing products were perceived as less effective. An interesting finding was that a specific pattern in effectiveness was observed for all product classes over the whole time of the study: either the product showed no effect at all or a moderate/strong effect. No product class appeared to be substantially superior to the others on an individual patient level. We therefore conclude that the best treatment option needs to be explored individually.

Only one in five patients received systemic antibiotics as primary prophylaxis from the beginning, so this drug class is often reserved for treatment-associated adverse events or as secondary prophylaxis. As expected, the frequency of acneiform rash grade ≥ 2 increased during the treatment course, from 12.6% at week 2 to 21.7% at week 16. These numbers are reassuring in the sense that the majority of patients did not develop severe skin reactions. Nevertheless, the proportion of patients whose skin reactions did impact on daily life steadily increased during treatment, reaching 76.7% at week 16. In line with this observation, the reactive use of all products tended to increase during the treatment course. These findings underline the importance of assessing not only severe toxicities, as also mild or moderate toxicities might affect QoL and patients’ daily life. The toxicity rates confirm published data from prospective clinical trials investigating cetuximab in patients with mCRC and r/m SCCHN.

The results of our study need to be discussed in the context of prior studies investigating different approaches of skin toxicity management in patients treated with cetuximab. In a US-American monocenter randomized controlled trial on 48 patients, prophylaxis with oral minocycline decreased the severity of acneiform rash during the first month of cetuximab treatment, while topical tazarotene was associated with significant irritation [12]. In the same institution, a prospective randomized trial of topical pimecrolimus for cetuximab-associated acne-like eruption in 24 patients failed to show clinically meaningful benefit [13]. Conversely, in a case series of 20 patients treated with different epidermal growth factor receptor inhibitors in Greece, pimecrolimus cream 1% (substituted by metronidazole 1% cream) was effective in most patients (> 50% reduction of erythema, pustules and pruritus) [14]. Minocycline was used in seven patients in the PROSKIN study, none was treated with topical tazarotene or pimecrolimus. In a study with historical controls as comparator, in 40 patients, cetuximab prophylaxis with topical vitamin K1 cream did not translate into clinically meaningful benefit in terms of reducing acneiform rash [15]. However, an Italian monocenter study of 41 patients with metastatic colorectal cancer treated with cetuximab suggested a possible benefit of topical vitamin K1-based cream as prophylaxis for skin rash [16]. In our PROSKIN study, most physicians perceived a moderate effect of topical vitamin K1 cream.

Ocvirk et al. reported on a group of 31 patients with acne-like rash, who, after the first documented cutaneous toxicity, received topical use of emollients. Patients with grade 2 rash received emollients and topical antibiotics. Patients developing grade 3 rash discontinued therapy with cetuximab until recovery and were treated with emollients, topical and systemic antibiotics. Of 31 patients, six had grade 3 rash, 16 patients grade 2 and nine patients grade 1 acne-like rash [17]. As in our study, no grade 4 skin reactions were observed.

A multicenter observational study in 55 patients reported delayed occurrence and milder course of skin reactions with the use of a pre-defined prophylactic skin care regimen including vitamin K1 ointment and oral doxycycline [17].

Overall, similar to our findings, results from previous studies show conflicting results and could so far not establish an evidence-based prophylaxis or treatment regimen for cetuximab-related skin toxicities. Nevertheless, the topic was discussed at consensus meetings and recommendations have been published [18, 19]. Another relevant factor is the low rate of consensus between oncologists and dermatologists in labeling and grading of skin reactions and the frequent interobserver variability [20].

A number of limitations and strengths of the study design need to be considered when discussing the results of this study. The study was small, had no control arm and was unblinded and thus should be considered as real-world observational study with focus on skin products used and their perceived effectiveness over time. In many subgroups, the number of patients was small and therefore results have to be carefully interpreted. There was a high rate of patients that were lost to follow-up. All patients came from Switzerland which had advantages in terms of the access of patients to products and uniform procedures, but might limit the generalizability of the findings to patients in other health-care settings and countries. It was the clinical decision of the respective treating physicians to assign selected patients to certain therapies and not to other available treatment options that potentially may introduce allocation or channeling bias and confound the association between treatment and outcomes [21]. However, this is an advantage as physicians already have some experience with the regimen they use. With a pre-defined regimen, there would be a learning curve at the beginning and possible skepticism that could influence how physicians perceive the effectiveness. Physicians and patients willing to participate in non-interventional studies such as ours may be particularly motivated or interested in science and therefore also be patient to selection bias. Finally, the follow-up period of 16 weeks was relatively short. However, it has been shown that skin toxicities usually develop within the first few weeks of cetuximab therapy. Therefore, we defined this time window. Moreover, at week 16 a large proportion of patients show tumor progression and these patients have to be discontinued from the study introducing another potential selection bias.

## Conclusions

In conclusion, in Switzerland oncologists used a great variety of low-cost general skin care products for the prevention or treatment of cetuximab-related skin toxicity in patients with mCRC or r/m SCCHN. They gave preference to systemic antibiotics and vitamin K1 cream. Patients perceived overall “moderate” efficacy of the various measures. For most patients, skin reactions did not influence their willingness to continue cetuximab therapy.

## Data Availability

The datasets generated and/or analyzed during the current study are not publicly available as sharing is not explicitly covered by patient consent. Medical History (FAS) *N *= 125
